# Bis(2-amino­pyrazine-κ*N* ^4^)dichlorido­zinc

**DOI:** 10.1107/S1600536811026031

**Published:** 2011-07-09

**Authors:** Shan Gao, Seik Weng Ng

**Affiliations:** a Key Laboratory of Functional Inorganic Material Chemistry, Ministry of Education, Heilongjiang University, Harbin 150080, People’s Republic of China; b Department of Chemistry, University of Malaya, 50603 Kuala Lumpur, Malaysia; c Chemistry Department, Faculty of Science, King Abdulaziz University, PO Box 80203 Jeddah, Saudi Arabia

## Abstract

In the title adduct, [ZnCl_2_(C_4_H_5_N_3_)_2_], the Zn^II^ atom lies on a twofold rotation axis that relates one Cl atom to the other as well as one 2-amino­pyrazine ligand to the other; the coordination geometry is a distorted tetra­hedron. In the crystal, adjacent mol­ecules are linked by N—H⋯N hydrogen bonds across the center of inversion, generating a chain; neighboring chains are linked by N—H⋯Cl hydrogen bonds, forming a three-dimensional network.

## Related literature

For a related compound, CoCl_2_(C_4_H_5_N_3_)_4_, see: Kang *et al.* (2009 ▶).
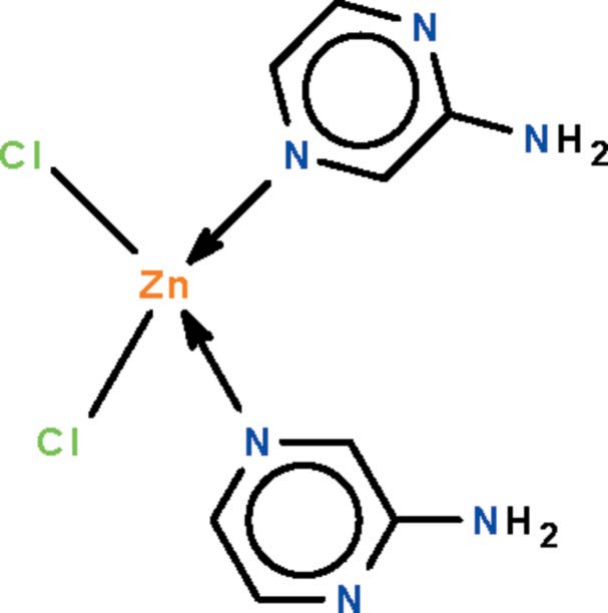


## Experimental

### 

#### Crystal data

[ZnCl_2_(C_4_H_5_N_3_)_2_]
*M* *_r_* = 326.49
Monoclinic, 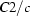

*a* = 17.1445 (12) Å
*b* = 6.1660 (4) Å
*c* = 12.0198 (8) Å
β = 98.608 (2)°
*V* = 1256.34 (15) Å^3^
*Z* = 4
Mo *K*α radiation
μ = 2.37 mm^−1^
*T* = 293 K
0.35 × 0.30 × 0.15 mm

#### Data collection

Rigaku R-AXIS RAPID IP diffractometer
Absorption correction: multi-scan (*ABSCOR*; Higashi, 1995 ▶) *T*_min_ = 0.491, *T*_max_ = 0.718
5769 measured reflections
1432 independent reflections
1350 reflections with *I* > 2σ(*I*)
*R*_int_ = 0.029

#### Refinement

*R*[*F* ^2^ > 2σ(*F* ^2^)] = 0.024
*wR*(*F* ^2^) = 0.066
*S* = 1.06
1432 reflections
84 parameters
2 restraints
H atoms treated by a mixture of independent and constrained refinement
Δρ_max_ = 0.32 e Å^−3^
Δρ_min_ = −0.32 e Å^−3^

### 

Data collection: *RAPID-AUTO* (Rigaku, 1998 ▶); cell refinement: *RAPID-AUTO*; data reduction: *CrystalClear* (Rigaku/MSC, 2002 ▶); program(s) used to solve structure: *SHELXS97* (Sheldrick, 2008 ▶); program(s) used to refine structure: *SHELXL97* (Sheldrick, 2008 ▶); molecular graphics: *X-SEED* (Barbour, 2001 ▶); software used to prepare material for publication: *publCIF* (Westrip, 2010 ▶).

## Supplementary Material

Crystal structure: contains datablock(s) global, I. DOI: 10.1107/S1600536811026031/xu5256sup1.cif

Structure factors: contains datablock(s) I. DOI: 10.1107/S1600536811026031/xu5256Isup2.hkl

Additional supplementary materials:  crystallographic information; 3D view; checkCIF report

## Figures and Tables

**Table 1:** Selected bond lengths (Å)

Zn1—N3	2.0576 (12)
Zn1—Cl1	2.2403 (4)

**Table 2:** Hydrogen-bond geometry (Å, °)

*D*—H⋯*A*	*D*—H	H⋯*A*	*D*⋯*A*	*D*—H⋯*A*
N1—H1⋯N2^i^	0.87 (1)	2.27 (1)	3.141 (2)	176 (3)
N1—H2⋯Cl1^ii^	0.87 (1)	2.63 (2)	3.392 (2)	147 (2)

Symmetry codes: (i) 
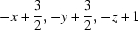
; (ii) 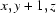.

**Fig. 1.**
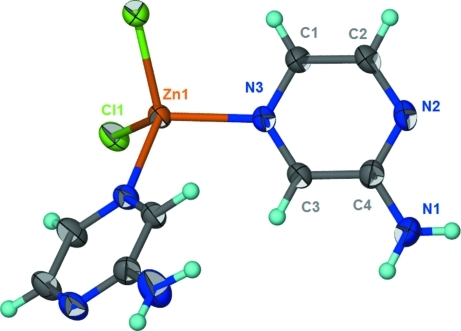
Thermal ellipsoid plot (Barbour, 2001) of ZnCl2(C4H5N3)2 at the 50% probability level; hydrogen atoms are drawn as spheres of arbitrary radius. The Zn atom lies on a twofold axis and the unlabeled atoms are related to the labeled ones by 1 - x, y, 1/2 - z.

## supplementary crystallographic information

### Comment

We have reported the metal(II) dichloride adducts of 2-aminopyrazine. For example, cobalt(II) dichloride forms a tetrakis adduct (Kang *et al.*, 2009). The corresponding zinc(II) dichloride is a bis adduct; in the adduct, ZnCl_2_(C_4_H_5_N_3_)_2_ (Scheme I, Fig. 1), the Zn^II^ atom lies on a twofold axis and the geometry is a tetrahedron. Adjacent adduct molecules are linked by an N–H···N hydrogen across a center-of-inversion to generate a chain; neighboring chains are linked by an N–H···Cl hydrogen bond to form a layer (Table 1).

### Experimental

Zinc dichloride hexahydrate (2 mmol) and 2-aminopyrazine (2 mmol) were dissolved in water (20 ml); the solution was filtered. Colorless crystals separated from solution after several days.

### Refinement

Carbon-bound H-atoms were placed in calculated positions (C–H 0.93 Å) and were included in the refinement in the riding model approximation, with *U*(H) set to 1.2*U*(C). The amino H-atoms were located in a difference Fourier map, and were refined with a distance restraint of N–H 0.88±0.01 Å; their temperature factors were refined.

### Crystal data

**Table:**

[ZnCl_2_(C_4_H_5_N_3_)_2_]	*F*(000) = 656
*M**_r_* = 326.49	*D*_x_ = 1.726 Mg m^−^^3^
Monoclinic, *C*2/*c*	Mo *K*α radiation, λ = 0.71073 Å
Hall symbol: -C 2yc	Cell parameters from 5413 reflections
*a* = 17.1445 (12) Å	θ = 3.4–27.5°
*b* = 6.1660 (4) Å	µ = 2.37 mm^−^^1^
*c* = 12.0198 (8) Å	*T* = 293 K
β = 98.608 (2)°	Prism, colorless
*V* = 1256.34 (15) Å^3^	0.35 × 0.30 × 0.15 mm
*Z* = 4	

### Data collection

**Table:**

Rigaku R-AXIS RAPID IP diffractometer	1432 independent reflections
Radiation source: fine-focus sealed tube	1350 reflections with *I* > 2σ(*I*)
graphite	*R*_int_ = 0.029
ω scans	θ_max_ = 27.5°, θ_min_ = 3.4°
Absorption correction: multi-scan (*ABSCOR*; Higashi, 1995)	*h* = −22→22
*T*_min_ = 0.491, *T*_max_ = 0.718	*k* = −7→7
5769 measured reflections	*l* = −15→15

### Refinement

**Table:**

Refinement on *F*^2^	Primary atom site location: structure-invariant direct methods
Least-squares matrix: full	Secondary atom site location: difference Fourier map
*R*[*F*^2^ > 2σ(*F*^2^)] = 0.024	Hydrogen site location: inferred from neighbouring sites
*wR*(*F*^2^) = 0.066	H atoms treated by a mixture of independent and constrained refinement
*S* = 1.06	*w* = 1/[σ^2^(*F*_o_^2^) + (0.0415*P*)^2^ + 0.3611*P*] where *P* = (*F*_o_^2^ + 2*F*_c_^2^)/3
1432 reflections	(Δ/σ)_max_ = 0.001
84 parameters	Δρ_max_ = 0.32 e Å^−^^3^
2 restraints	Δρ_min_ = −0.32 e Å^−^^3^

### Fractional atomic coordinates and isotropic or equivalent isotropic displacement parameters (Å^2^)

**Table:**

	*x*	*y*	*z*	*U*_iso_*/*U*_eq_	
Zn1	0.5000	0.04567 (4)	0.2500	0.03073 (11)	
Cl1	0.44416 (2)	−0.15067 (7)	0.37370 (3)	0.04100 (13)	
N1	0.63303 (10)	0.7312 (3)	0.48482 (17)	0.0590 (5)	
H1	0.6731 (12)	0.808 (4)	0.516 (2)	0.088*	
H2	0.5858 (9)	0.779 (5)	0.486 (2)	0.088*	
N2	0.71831 (9)	0.4959 (3)	0.41464 (14)	0.0431 (3)	
N3	0.59049 (7)	0.2453 (2)	0.31966 (11)	0.0330 (3)	
C1	0.66520 (10)	0.1889 (3)	0.30962 (16)	0.0439 (4)	
H1A	0.6746	0.0646	0.2698	0.053*	
C2	0.72731 (10)	0.3139 (3)	0.35775 (17)	0.0456 (4)	
H2A	0.7782	0.2698	0.3504	0.055*	
C3	0.57962 (10)	0.4231 (3)	0.37660 (14)	0.0352 (3)	
H3	0.5287	0.4646	0.3850	0.042*	
C4	0.64450 (10)	0.5519 (2)	0.42521 (15)	0.0380 (4)	

### Atomic displacement parameters (Å^2^)

**Table:**

	*U*^11^	*U*^22^	*U*^33^	*U*^12^	*U*^13^	*U*^23^
Zn1	0.02308 (15)	0.02663 (16)	0.04198 (17)	0.000	0.00323 (10)	0.000
Cl1	0.0322 (2)	0.0426 (2)	0.0489 (2)	0.00146 (16)	0.00822 (16)	0.01054 (17)
N1	0.0411 (9)	0.0467 (9)	0.0853 (12)	−0.0010 (8)	−0.0031 (8)	−0.0283 (9)
N2	0.0292 (7)	0.0403 (7)	0.0577 (9)	−0.0066 (6)	−0.0007 (6)	−0.0060 (7)
N3	0.0265 (6)	0.0298 (6)	0.0422 (6)	−0.0028 (5)	0.0035 (5)	−0.0027 (5)
C1	0.0301 (8)	0.0404 (9)	0.0617 (10)	−0.0027 (7)	0.0086 (7)	−0.0139 (8)
C2	0.0257 (8)	0.0477 (9)	0.0636 (11)	−0.0030 (7)	0.0070 (7)	−0.0081 (8)
C3	0.0261 (8)	0.0330 (7)	0.0460 (8)	0.0003 (6)	0.0034 (6)	−0.0016 (6)
C4	0.0350 (9)	0.0320 (8)	0.0450 (9)	−0.0022 (6)	−0.0008 (7)	−0.0023 (6)

### Geometric parameters (Å, °)

**Table:**

Zn1—N3	2.0576 (12)	N2—C4	1.336 (2)
Zn1—N3^i^	2.0576 (12)	N3—C3	1.320 (2)
Zn1—Cl1	2.2403 (4)	N3—C1	1.350 (2)
Zn1—Cl1^i^	2.2403 (4)	C1—C2	1.371 (2)
N1—C4	1.348 (2)	C1—H1A	0.9300
N1—H1	0.87 (1)	C2—H2A	0.9300
N1—H2	0.87 (1)	C3—C4	1.419 (2)
N2—C2	1.335 (2)	C3—H3	0.9300
			
N3—Zn1—N3^i^	106.52 (7)	N3—C1—C2	120.33 (16)
N3—Zn1—Cl1	115.10 (4)	N3—C1—H1A	119.8
N3^i^—Zn1—Cl1	102.85 (4)	C2—C1—H1A	119.8
N3—Zn1—Cl1^i^	102.85 (4)	N2—C2—C1	123.16 (17)
N3^i^—Zn1—Cl1^i^	115.10 (4)	N2—C2—H2A	118.4
Cl1—Zn1—Cl1^i^	114.58 (2)	C1—C2—H2A	118.4
C4—N1—H1	120.5 (19)	N3—C3—C4	121.01 (15)
C4—N1—H2	120 (2)	N3—C3—H3	119.5
H1—N1—H2	119 (3)	C4—C3—H3	119.5
C2—N2—C4	116.72 (15)	N2—C4—N1	118.57 (16)
C3—N3—C1	118.01 (14)	N2—C4—C3	120.76 (15)
C3—N3—Zn1	123.46 (11)	N1—C4—C3	120.67 (17)
C1—N3—Zn1	118.50 (10)		
			
N3^i^—Zn1—N3—C3	41.52 (11)	C4—N2—C2—C1	1.2 (3)
Cl1—Zn1—N3—C3	−71.75 (13)	N3—C1—C2—N2	−1.0 (3)
Cl1^i^—Zn1—N3—C3	162.95 (12)	C1—N3—C3—C4	0.4 (2)
N3^i^—Zn1—N3—C1	−140.45 (14)	Zn1—N3—C3—C4	178.43 (12)
Cl1—Zn1—N3—C1	106.28 (13)	C2—N2—C4—N1	178.12 (19)
Cl1^i^—Zn1—N3—C1	−19.02 (13)	C2—N2—C4—C3	−0.7 (3)
C3—N3—C1—C2	0.1 (3)	N3—C3—C4—N2	−0.1 (3)
Zn1—N3—C1—C2	−178.02 (15)	N3—C3—C4—N1	−178.88 (17)

Symmetry codes: (i) −*x*+1, *y*, −*z*+1/2.

### Hydrogen-bond geometry (Å, °)

**Table:**

*D*—H···*A*	*D*—H	H···*A*	*D*···*A*	*D*—H···*A*
N1—H1···N2^ii^	0.87 (1)	2.27 (1)	3.141 (2)	176 (3)
N1—H2···Cl1^iii^	0.87 (1)	2.63 (2)	3.392 (2)	147 (2)

Symmetry codes: (ii) −*x*+3/2, −*y*+3/2, −*z*+1; (iii) *x*, *y*+1, *z*.

## References

[bb1] Barbour, L. J. (2001). *J. Supramol. Chem.* **1**, 189–191.

[bb2] Higashi, T. (1995). *ABSCOR* Rigaku Corporation, Tokyo, Japan.

[bb3] Kang, W., Huo, L.-H., Gao, S. & Ng, S. W. (2009). *Acta Cryst.* E**65**, m1502. 10.1107/S1600536809045309 PMC2972178 21578553

[bb4] Rigaku (1998). *RAPID-AUTO* Rigaku Corporation, Tokyo, Japan.

[bb5] Rigaku/MSC (2002). *CrystalClear* Rigaku/MSC Inc., The Woodlands, Texas, USA.

[bb6] Sheldrick, G. M. (2008). *Acta Cryst.* A**64**, 112–122. 10.1107/S0108767307043930 18156677

[bb7] Westrip, S. P. (2010). *J. Appl. Cryst.* **43**, 920–925.

